# The Diagnosis of “Pervasive Developmental Disorder Not Otherwise Specified”: A Systematic Literature Review

**DOI:** 10.3390/children10050844

**Published:** 2023-05-07

**Authors:** Agostino Carbone, Asia Dell’Aquila

**Affiliations:** Department of Developmental and Social Psychology, Faculty of Medicine and Psychology, Sapienza University of Rome, 00185 Roma, Italy

**Keywords:** diagnostic process, PDD-NOS, autism spectrum disorder, DSM, neuropsychiatry of childhood, clinical psychology

## Abstract

(1) Background: The study deepens the diagnosis of “pervasive developmental disorder not otherwise specified” (PDD-NOS), a subthreshold diagnosis introduced in DSM-IV and then absorbed into the broader spectrum disorder of autism in DSM 5. The presence of people still attributed with a diagnosis of PDD-NOS can cause confusion in the understanding of this disorder, which is no longer present in the current diagnostic system. This review aims to gain a deeper understanding of the characteristics and boundaries of diagnosis, its use within the scientific community, and the long-term stability of that diagnosis. (2) Methods: The Prisma method was used to carry out the literature review; the scientific papers were selected using scientific search engines, including SCOPUS, PUBMED, and PsychINFO. Twenty-three articles were finally selected, and a meticulous reading was carried out in relation to the research questions. (3) Results: Four cross-cutting categories were identified: (1) diagnosis; (2) differential diagnosis; (3) prognosis; and (4) comorbidity. (4) Conclusions: Limits have emerged regarding the consistency, sensitivity, and the stability of PDD-NOS. The inclusion of this diagnosis within the broader autism spectrum disorder category coined in DSM-5 appears applicable.

## 1. Introduction

The study deals with Pervasive Developmental Disorder Not Otherwise Specified (PDD-NOS), a diagnosis introduced in the Diagnostic and Statistical Manual of Mental Health Disorders 4th edition (DSM-IV) [1] and that the DSM-5 [2] eliminated by including it in the Autism Spectrum Disorders, a category that has incorporated the DSM-IV diagnoses of Autistic Disorder and Asperger’s Syndrome. This was a choice owing to the high comorbidity rate of PDD-NOS with these diagnoses; after all, psychiatric comorbidities in children with autism spectrum disorder are a norm rather than an exception [3].

Until its inclusion in autism spectrum diagnosis, PDD-NOS was used as a subthreshold diagnosis of autism disorder. This has generated a share of confusion both within the professional sphere and within the caregivers of children diagnosed with PDD-NOS, who were relieved that they had not been diagnosed with an autistic disorder, considered by common sense often as a “serious” diagnosis. The inclusion of the diagnosis of PDD-NOS within the “new” autism spectrum diagnosis in 2013 had several consequences that generated considerable ambiguity, uncertainty, and unreliability in the diagnostic process and in the classification systems.

Specifically, the biggest problem is making sense of non-intuitive concepts such as “subthreshold diagnosis” of “autism spectrum”. The purpose of this systematic review of the literature is to shed light on this diagnostic transition, collecting and interpreting the considerations of the scientific world on the problems of reliability and validity of the diagnosis of PDD-NOS and the considerations that led to the inclusion of PDD-NOS within an umbrella diagnostic category definable as the “spectrum” of autistic disorder.

The specific aim of that paper is to gain a deeper understanding of the characteristics and boundaries of that diagnosis of pervasive developmental disorder not otherwise specified, its use within the scientific community, and the prognosis of children and adolescents to whom the diagnosis has been attributed.

### PDD-NOS Journey in the Classification Systems

In the International Classification of Diseases, ICD-10 [4] in Chapter V, Mental and Behavioral Pathologies, we find F80-F89 psychological development disorders, in which we can find under the specific code F84 pervasive developmental disorders. They are described as a group of disorders that is characterized by qualitative impairments in social interactions and modes of communication and by a limited, stereotyped, and repetitive repertoire of interests and activities. Among the four sub-categories, we find code F84.9 “pervasive developmental disorder, unspecified”.

The various changes that this diagnosis has undergone within the psychiatric classification, the ambiguity, and vagueness with which the diagnosis is treated in the various nosography systems, together with scarce longitudinal scientific research, have made it difficult to appreciate (1) its peculiarities within the diagnostic system, (2) its use within the professional community, and (3) a stable ability to predetermine the functional evolution of the people to whom the diagnosis has been attributed.

In fact, the absence of defined criteria can create anxiety and confusion in the family to which the children belong, and also creates difficulties in knowing and choosing the most effective intervention and strategies regarding their wellness and wellbeing. For these reasons, it is necessary to study this problem in depth.

This paper introduces a critical reading and comprehension of the diagnostic criteria inherent to pervasive developmental disorder not otherwise specified using a systematic review of the scientific literature. The aim of the study is to define the comorbidities, the specificity, and sensibility of pervasive developmental disorder not otherwise specified and the prognosis over a medium to long time span. Pervasive developmental disorder not otherwise specified (PDD-NOS) falls under the diagnostic category of pervasive developmental disorders (PDD), a group of disorders that is characterized by delays in the development of different basic functions, including communication and socialization. Autism, Asperger’s syndrome, childhood disintegrative disorder (CDD), hyperactive disorder associated with mental retardation and stereotyped movements, and Rett syndrome are also included in this category.

Pervasive developmental disorder not otherwise specified is one of the conditions that is part of the autism spectrum disorders, resulting in severe and generalized impairment in the development of reciprocal social interaction associated with impaired verbal or non-verbal communication skills and the presence of stereotyped behavior, interests, or activities.

Autism spectrum disorders (ASD) include autistic disorder, Asperger’s syndrome, and generalized developmental disorder not otherwise specified.

This disorder has historically been used to describe individuals who did not fully meet the specific criteria for autism, Asperger’s syndrome, schizophrenia, schizotypal personality disorder, or avoidant personality disorder. In pervasive developmental disorder not otherwise specified, autism-typical was included to indicate a global disruption of the personality, with disharmonious relational and cognitive abilities, which was severely impaired in some areas and intact in others. Most studies on samples of children [5,6,7] agree that the majority of individuals with pervasive developmental disorder not otherwise specified turn out to have many more deficits on the communication side.

The disorder is described in different ways in different diagnostic manuals.

In the latest edition of the DSM-5 [2], there is a new designation of autism spectrum disorders, which includes autistic disorder, Asperger’s disorder, and DGS-NAS (generalized developmental disorder not otherwise specified).

In the DSM-5 [2], we find the new diagnostic classification of ASD:

(1) the reduction of symptom domains from three to two (socio-communicative deficits and stereotyped interests and/or repetitive behavior); (2) the indication of the level of severity of the symptoms manifested within these two domains, which is done on a three-point scale.

In the Diagnostic Classification of Mental Health and Developmental Disorders of Infancy and Early Childhood: DC: 0–5, the diagnosis of PDD-NOS may correspond to the diagnoses of Early Atypical Autism Spectrum Disorder or of Global Developmental Delay, diagnosed when the child presents a significantly below-average level of functioning in all developmental domains.

Starting from the construction of the “Autism Spectrum Disorder” diagnostic category by the DSM-5 classification system in 2013, the system which, as previously mentioned, has absorbed all the pervasive developmental disorders, similar to the other classification systems. Starting from the creation of the “autism spectrum” category by the DSM-5 classification system, which, as previously mentioned, has absorbed all the pervasive developmental disorders, the other classification systems, ICD-11 and DC 0–5, complied with this proposal of the American Psychiatric Association, using the broader diagnosis of “Autism Spectrum Disorder” and incorporating into it all related diagnoses similar to PPDs.

## 2. Rationale

The research is a systematic review of the scientific literature regarding the diagnosis of pervasive developmental disorder not otherwise specified. The aim is to gain a deeper understanding of the characteristics and boundaries of the diagnosis of pervasive developmental disorder not otherwise specified, its use within the scientific community, and the prognosis of children and adolescents to whom the diagnosis has been attributed.

This systematic review can play many essential roles. It can provide summaries of the state of knowledge in an area, from which future research priorities can be identified; it can address issues that otherwise could not be resolved by individual studies; it can identify problems in primary research that should be resolved in future studies; and it can generate or evaluate theories about how or why phenomena occur. In sum, this systematic review thus generates different types of knowledge for different users, scholars, as well as clinicians.

## 3. Methods

**Figure 1:**
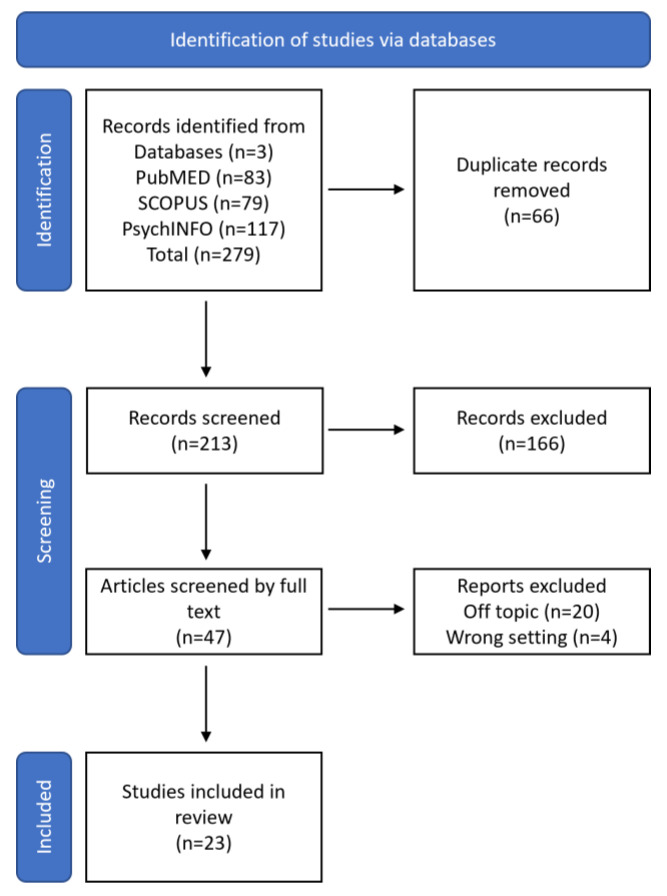
Flow-chart.

The PRISMA method was used to carry out a literature review [8,9]; it is designed primarily for the systematic review of studies assessing the effects of health interventions. Comprehensive reporting allows readers to assess the appropriateness of the methods and thus the reliability of the results. Presenting and summarizing the characteristics of studies that contribute to a synthesis allows health professionals and policymakers to assess the applicability of the results to their context. It helps researchers in the process of writing the paper and emphasizing the essential aspects without any possibility of forgetting or approximating. PRISMA contains two basic elements: (a) the flow-chart (Figure 1), which graphically describes the process of screening, selection, and inclusion of articles; (b) a 27-item checklist aimed at guiding the authors in an optimal and transparent description of the whole paper from the title to the conclusions.

### Procedures

The present systematic review was carried out using the principles of the PRISMA and the International Prospective Register of Systematic Review Registry guidelines (PROSPERO ID No. 410045).

The Scopus, PubMed, and PsychINFO were used to find papers matching out topic dating from 1 January 2010 up to January 2023, with English-language restriction. These search engines allow the identification of texts from the academic literature using specific keywords. They make it possible to find articles from a wide range of publishers and journals. The keywords used to search for articles related to the thesis topic were: “pervasive developmental disorder not otherwise specified”, “PDD-NOS”, “asperger”, “diagnosis” in the “Title/Abstract”. The Boolean approach was used by means of OR, AND, NOT operators (Table 1). The whole procedure is described in detail step by step in the flow chat diagram (Figure 1). The research group is made up of a junior researcher and two senior researchers, one of which is an expert in IT processes and an expert in diagnostic processes and disability psychology, a professional researcher, and an expert in psychodynamic processes and systematic literature reviews.

Titles and complete texts of some papers were qualitatively evaluated according to its topicality. In the next step, duplicates were eliminated and the outcomes were screened qualitatively through inclusion/exclusion criteria created for this purpose. The various changes that this diagnosis has undergone within the psychiatric classification, the ambiguity, and vagueness with which the diagnosis is treated in the various nosography systems, together with scarce longitudinal scientific research, have made it difficult to appreciate (1) its peculiarities within the diagnostic system, (2) its use within the professional community, and (3) a stable ability to predetermine the functional evolution of the people to whom the diagnosis has been attributed.

All appropriate studies were analyzed by means of the following inclusion criteria: chapter of books and scientific articles focusing specifically on the topic of PDD-NOS and not only tangentially; studies published in English language; studies or research with a sample under the age of 18 years; and systematic reviews of the literature reviews of reviews, case reports, randomized trials, retrospective studies, and observational studies. Otherwise, the exclusion criteria were as follows: no comparison between PDD-NOS and autism spectrum disorder; genetic studies; studies on physical symptoms; studies on the correlation between autism and vaccines; studies of abnormalities between biochemical imbalances and autism spectrum syndromes; functional imaging-based clinical trials (fMRI); studies in which the reference sample is over 18 years of age; and local studies.

The research through the search engines was implemented by three members of the research group, with the exception of the professorial researcher who joined the process in the following phase, offering his advice in carrying out the qualitative screening of the articles by sharing and re-discussing the criteria of inclusion and exclusion. Agreement among the researchers was reached through several consensus meetings, which ensured the independence of the researchers and the reliability of the results.

## 4. Results

A total of 279 publications were detected from the subsequent databases: Scopus (79), PubMed (83) and PsychINFO (117). Subsequently, 66 duplicates were located and removed. The remaining 213 records were selected through a careful reading and analysis of the abstracts, titles and keywords; at the end of this screening process, 166 articles were rejected because they were not relevant to the research question (the keywords used in search engines did not reflect the centrality of records). In some cases, they were comments, letter to editors, or conference proceedings, while in other cases, they covered related situational studies whose results were linked to characteristics of the local culture; in other cases, some were discarded due to repetition of topics or the lack of coherence.

In the final phase, the remaining 47 papers were screened by reading the full text; 24 were excluded because 4 were off-topic and 4 included the wrong settings. The entire screening process is described and summarized in detail in Figure 1.

A final number of 23 studies were included in the review for the interpretative analysis and the identification and discussion of emerging themes through sharing meetings between researchers (Table 2).

The systematic review of the scientific literature produced four emerging themes: (1) the characteristics of the diagnosis and the cases in which it is assigned; (2) differential diagnosis; (3) prognosis; and (4) comorbidity. The categories were identified by the principal investigator and then discussed with a senior researcher.

## 5. Discussion

### 5.1. Fist Theme—Characteristics of the Diagnosis and Cases in Which It Is Assigned

The first topic emerged concerns the characteristics of this diagnosis and the different cases in which it is assigned. The DSM-IV includes definitions of four other disorders in the category of pervasive developmental disorders, namely (1) Asperger’s disorder, (2) Rett’s disorder, (3) childhood disintegrative disorder, and (4) the residual category of pervasive developmental disorder not otherwise specified.

PDD-NOS is described as a subthreshold category for those disorders that are similar to autism, but do not meet the full set of criteria for the condition. Because no algorithm or threshold has been specified in the definition, both research and clinical practice are hampered by the absence of clear guidelines to adopt. This sub-threshold or residual diagnosis appears to be used in four different ways:

(1) the case is a variant of autistic disorder, i.e., fewer criteria are met than for autism;
(2) the case is characterized by uncommon and possibly idiosyncratic autistic symptoms;
(3) the case fails to meet the specified criteria patterns (e.g., less than two social symptoms or no symptoms in the communication or activity/interest domains);
(4) the case fails to meet the criterion for the onset of autism.

The number of cases meeting the DSM-IV definition of PDD-NOS should be lower than the number of PDD-NOS cases as defined in DSM-III-R, considering that DSM-IV includes three additional pervasive developmental disorders.

A study carried out by researchers [5] pointed out that with the advent of the DSM-5, the subcategories of pervasive developmental disorders were eliminated and replaced with the single diagnosis of autism spectrum disorder (ASD). The DSM-IV characterized individuals diagnosed with an ASD on the basis of behavioral characteristics in three domains: social reciprocity, communication, and restricted or stereotyped behaviors or interests [1] The DSM-5, on the other hand, reduced the domains from three to two: social reciprocity and restricted or stereotyped behaviors or interests [2].

Two of the three ASDs, autistic disorder and Asperger’s syndrome, had diagnostic criteria that were outlined in the DSM-IV-TR. The behaviors needed for the diagnosis were not explicitly specified in the PDD-NOS diagnostic criteria. Instead, PDD-NOS was diagnosed in children who showed a variety of symptoms, such as impaired social interaction skills, communication issues, and the presence of repetitive or stereotypical behaviors. Therefore, despite criticism, PDD-NOS has continued to be a highly prevalent diagnosis; it has been assigned at 1.7 times the rate of autistic disorders. Pervasive developmental disorder that is not otherwise specified is described as potentially problematic and lacks explicit operational definitions due to poor inter-rater reliability. There is probably some heterogeneity in this population because there are no other more precise criteria for a PDD-NOS diagnosis.

There have been few studies that have attempted to further specify the diagnosis of PDD-NOS [10,11,12,13]. Research has mainly described pervasive developmental disorder not otherwise specified in relation to other ASDs with the aim of examining whether each disorder had unique and differentiated profiles or whether each diagnosis differed only in their position along a spectrum of symptom severity [20].

In the past, it was intended to clarify some of the heterogeneity in each disorder’s presentations and thereby the etiology and trajectory of each by validating subgroups under the larger umbrella of pervasive developmental disorders. ASD diagnoses are thought to represent a range of symptom severity, according to several studies. This conceptualization, now accepted by the DSM-5, makes the case that the severity of a child’s autism-related symptoms is the only factor determining how different each disorder is. Therefore, according to this interpretation, the profile of PDD-NOS is not qualitatively distinct from that of other ASDs. 

The PDD-NOS profile might not perfectly fit along the spectrum of symptom severity, so it would differ from other ASDs, according to a second viewpoint. According to a study, children with pervasive developmental disorder—which is not otherwise identified—have histories of age delays that are unusual for Asperger’s syndrome and exhibit stereotyped and repetitive behavior less frequently than those with the disorder.

Several studies in the first ten years of the 2000s questioned whether the PDD-NOS category was appropriate. The literature has not yet been able to clearly distinguish between children with PDD-NOS and those with AD [16], despite the fact that kids with this disorder exhibited less repetitive and constrictive behavior than kids who had autism. Instead of being correlated with autistic symptoms, distinctions have been found to be inconsistent and linked to cognitive function and language level.

The validity of the current symptom domains and subtypes has been questioned in a number of studies, highlighting the need for more empirical conceptualization. The proposal to combine the PDD subtypes into a single category of autism spectrum disorders (ASD) in the DSM- 5 was motivated by the evidence that they cannot be reliably distinguished from one another and are comparable in terms of prognosis and treatment need. Finally, in addition to what has been said so far, longitudinal studies have further highlighted the impossibility that PDD subtypes can be reliably distinguished from each other, confirming the overall validity of the domain of ASD diagnosis [24].

### 5.2. Second Theme—Differential Diagnosis

The second theme concerns the procedure based on comparing the individual’s reported signs and symptoms to arrive at a correct diagnosis, avoiding possible errors of assessment [17].

Pervasive developmental disorder not otherwise specified is a term commonly used in cases in which, although disturbances referable to social interaction, communication and a repertoire of restricted and stereotyped interests and activities are present, the clinical picture does not assume qualitatively defined and quantitatively sufficient characteristics for a diagnosis of autism or other DGS. The concept DGS-NAS describes morbid conditions in which the degree of impairment in one of the three central areas of deficit is rather slight compared to the autistic disorder. It is, therefore, a group of disorders in which the impairment in one or more areas is too moderate to allow the assignment of a severe diagnosis, yet at the same time, the seriousness of the deficit and the limitations are so strong as to exclude that the condition can be considered as a simple variant of normality.

The heterogeneous clinical entities that comprise ASD-NAS share three main characteristics: early onset of symptoms, impaired interpersonal relationships and impaired communication. Many authors have questioned, through comorbidity studies, whether significantly distinct groups exist and whether some individuals should be separated into other diagnostic categories that show phenotypic overlap with ASD, such as the proposed category of social communication disorder [18]; this study highlighted that the distinguishing feature of PDD-NOS is a greater impairment of communication skills compared to the diagnosis of ASD. There are no answers to these questions due to the sparse scientific literature and the few studies conducted over the years. It would be desirable that an update of the diagnostic system be suggested within the healthcare system, in particular regarding the diagnoses of the autism spectrum, which modifies and includes diagnoses that are no longer subject to differentiation within the diagnostic classification systems.

The feature common to several epidemiological studies, which most strongly differentiates subjects diagnosed with pervasive developmental disorder not otherwise specified from those diagnosed with autistic disorder, is that the former are found to have less restricted behavior and limited interests than the latter and greater language deficits.

The communication deficits of PDD-NOS and ASDs are generally expressed in the absence of communication skills (including non-verbal communication), to the extent that the subject may lack the facial expressions and mimicry necessary for a minimum of expressiveness towards the outside world. Abnormal non-verbal communication is present, such as the lack of eye contact. In addition, there is an absence of age-appropriate imaginative activity and abnormalities in verbal production, which may relate to rhythm, cadence, force, volume, tone or the form and content of speech.

One of the cases in which the diagnosis of PDD-NOS was misattributed instead of a diagnosis of AD was in cases of early diagnosis, particularly when at the time of the diagnostic examination, no stereotyped behaviors had appeared, ruling out a diagnosis of AD. However, it may happen that the stereotypical behaviors make their debut after the child turns 3 years, thus saturating the criteria for the attribution of AD.

Approximately half of the individuals diagnosed with ASD do not develop a way to express their basic needs; this deficit combined with the increasing number of diagnoses of autism spectrum disorders has led to a pressing need to identify evidence-based practices, especially for those who cannot use traditional means of communication.

In the following paragraphs, the meaning of this will be explained more clearly.

### 5.3. Third Theme—Prognosis

The development of children with pervasive developmental disorders that are not otherwise specified and their evolution are the subjects of the third theme. Children with PDD-NOS were found to have a higher chance of achieving the best outcomes as adults than those with other ASD diagnoses. Children with an ASD diagnosis at a young age who later re-evaluate their development and no longer show symptoms of the condition are referred to as having an “optimal outcome.” According to a study [24], 39% of the 11 children in their sample who were diagnosed with pervasive developmental disorder at around 2 years of age no longer met the ASD criteria by the time they were 4 years old. Children with PDD-NOS had a significantly higher rate of achievement than those who had been given an autistic disorder diagnosis at 11% [12].

A study [12] that looked at the diagnostic results of children with pervasive developmental disorder who had not previously been diagnosed at age 2 and were revisited at age 4 found that a number of variables predicted the greatest outcomes by that age. These included having fewer repetitive behaviors, having higher adaptive skills as judged by parental reports, and having higher expressive language skills. The children’s motor abilities were better early on in development, according to parents as well. By identifying kid subgroups within the DSM-IV-TR PDD-NOS, we may be better able to identify, understand, and serve these children. It may also provide pertinent information about a group of children whose ASD diagnoses may change in light of the revised DSM- 5 criteria.

The study’s goal was to examine the characteristics of a sample of young children with PDD-NOS who were about two years old. The study’s specific aim is to find more homogeneous and clinically significant subgroups within a sample of children with pervasive developmental disorder who are not otherwise characterized in order to increase the predictive value of future diagnoses. The research team hypothesized that, at age two, the features of the sub-groups revealed by cluster analysis would display an atypical profile, meaning that children in each subsubgroup would offer an account that differed in several areas rather than along the scale of symptom severity. In response to the popular perception that PDD-NOS is a diagnostic that covers all bases, this prediction was created (catchall).

At age 2, children with PDD-NOS may display behavioral patterns that hint at their likely developmental trajectory. Therefore, it was predicted that subgroup membership at age 2 and diagnostic outcome at age 4 would be related. Participants were selected from a larger sample of children to participate in an ongoing study examining the effectiveness of screening questionnaires designed to detect ASD symptoms in young children. Reassessments were conducted on fifteen of the children in cluster 1. The majority of the children in this cluster (*n* = 9, 60%) no longer satisfied the criteria for an ASD, whereas the other six children (40%) continued to get a stable PDD-NOS diagnosis. Five of the children in the study who no longer satisfied the requirements for an ASD had no diagnosis (33.3%), three had developmental delays (DD) (20%), and one had developmental language disorder (DLD) (6.7%).

At the age of 4, 38 children in cluster 2 underwent a second evaluation. The majority of them still met the requirements for PDD-NOS (*n* = 15, 39%) or those for an autistic disorder diagnosis at age 4 (*n* = 19, 50%). At the age of 4 years old, only 4% of the children (*n* = 11) did not exhibit ASD symptoms. Two of these kids (5.3%) had DD diagnoses, one had a DLD diagnoses (2.6%), and one had “no diagnosis” (2.6%). Despite having only a few repetitive behaviors at age 2 in cluster 3, the majority of the five children in this cluster who underwent re-evaluation at age 4 remained to meet the criteria for autism disorder (*n* = 3, 60%). At age 4, one child with a DD diagnosis ceased to meet the criteria for an ASD, while the other child was still meeting the PDD-NOS requirements.

Children in Cluster 1 supported previous research that suggested PDD-NOS patients may experience “better” results later in life (e.g., no longer fitting the criteria for an ASD). These kids had better scores on tests of nonverbal problem-solving, display less social interaction issues, have fewer repetitive habits, and have milder autism symptomatology [21].

By age 4, children in cluster 2 predicted either the maintenance of the PDD-NOS diagnosis or the emergence of a more severe diagnosis of autism disorder based on lower cognitive scores, greater social impairment, more frequent repetitive behaviors, and more extreme autism symptoms.

Clusters 1 and 2 might imply that the PDD-NOS population is less heterogeneous than what has been described in past work.

These results may suggest that some children who are diagnosed with PDD-NOS at age 2 appear to have a pattern of symptoms that is reasonably consistent, despite the lack of clearly defined criteria, even though the severity of this pattern varies greatly.

Last but not least, the data raise the possibility that children in cluster 1 may be labeled with socio-pragmatic communication disorder rather than ASD if they do not match the DSM- 5 criteria for an ASD diagnosis. The DSM- 5 criteria for an ASD diagnosis may not be met by young children if they do not exhibit stereotypical and repetitive behaviors or interests. Future researchers must continue to comprehend the trajectories of kids who do not show consistent repetitive and stereotypical behaviors by the age of two in order to ensure that kids with significant social and communication delays can get the crucial early intervention services for autism. Limiting access to these therapies when autistic symptoms are minimal or moderately present, as demonstrated in clusters 1 and 3, is likely to have a major impact on kids’ results at age 4. However, more investigation will be needed to support this assertion.

### 5.4. Forth Theme—Comorbidity

The fourth topic is comorbidity, or the presence of many diseases in the same person. Since individuals on the autism spectrum vary not only in their level and type of core autistic traits and cognitive abilities [22], but also in terms of concomitant behavioral and emotional problems [19,23], researchers Kirstin Greaves—Lord et al. [6] in a 2012 study of empirically based phenotypic profiles of children with PDD—NOS, found it useful to empirically consider all these characteristics from a dimensional perspective. Tracking behavioral and emotional issues concurrently can help us determine whether people on the “less extreme end of the autism spectrum” have more or less clinical needs than those with full ASD, which is important for determining whether they should be eligible for a diagnosis and/or services. Behavioral and emotional issues can have a significant impact on daily functioning [28]. The aforementioned details on comorbidities also help to clarify whether there are any significantly different groups and whether some people need to be classified under different diagnostic groups that exhibit phenotypic overlap with ASD, such as the proposed category of social communication disorder [14] or attention deficit hyperactivity disorder (ADHD).

The study aims to provide new empirical insights into the wide range of phenotypic traits of individuals diagnosed with PDD in an effort to advance the discussion over whether a DSM- 5 diagnosis of ASD is suitable for some persons or whether alternatives may be more appropriate.

The sample consists of 949 individuals with a PDD diagnosis according to the DSM-IV-TR between the ages of 6 and 18 (82 percent male, mean age 9.3). The Child Behavior Checklist, which was designed to assess comorbidity with behavioral and emotional disorders, was completed by the mothers of the children. The CBCL has 118 problem items, each of which is rated on a three-point scale. These traits—anxious/depressed, withdrawn, somatic complications, social problems, thinking/attention challenges, violent conduct, and aggressive behavior—are categorized using eight scientifically supported syndrome scores.

However, the parents of the children used the Children’s Social Behavior Questionnaire (CSBQ) to assess the wide range of autistic traits present in PDD children. This measure was developed to assess the numerous problem dimensions on which children with pervasive developmental disorders tend to differ in order to address the variability in this group. During the instrument’s development, the most severe autistic behaviors and the mildest PDD score distribution were combined to cover the full spectrum of autism. We tested if classes with distinct profiles of autistic features could be found using latent profile analysis (LPA), a technique that investigates whether it is possible to identify groups with comparable reactions on syllables. The primary objective of this study is to identify the fewest classes of individuals with various approval patterns.

According to research on behavioral and emotional issues, Grade 1 students generally performed well across the board, with particularly strong results in the areas of thinking, attention, and social issues. Grade 2 showed generally high ratings for all comorbidities, although there was more variance between behavioral and emotional problems than there was in grade 1, with behavioral issues being comparatively more severe. Social issues and thinking issues are both high in the second grade, with no significant differences on the majority of scales. Grade 3 mostly mirrors Grade 1 in terms of behavioral and emotional issues, with only minor variations in attention issues and social and emotional issues as well as average differences in reaction and communication issues. The majority of the emotional and behavioral issues in grade 4 are mild and fairly equal, in contrast to other grades where behavioral issues are more severe than emotional issues. Grade 5 comparisons show that similarities between Grades 1 and 6 exist.

The study discovered six classes with distinctive phenotypic features. The phenotypic features of three of these classes—4, 5, and 6—were not entirely consistent with how ASD is conceptualized in the DSM-5. The profiles of the other three classes (classes 1, 2, and 3) resembled those of ASD as it is conceptualized in the DSM-5. The first three kinds of people are intellectually capable and have an Asperger’s or PDD-NOS diagnosis. The authors would want to draw attention to the fact that when examining these people’s phenotypic characteristics in further detail, other diagnoses may be taken into account. For instance, class 5 exhibited social communication issues primarily rather than restricted and repetitive behaviors; in this situation, the new classification of social communication disorder (SCD) may be a good fit given the mild difficulty in comprehending pragmatic communication.

## 6. Conclusions

The transition from DSM-IV to DSM-5 has had many consequences within diagnostic classifications, in particular with regard to neurodevelopmental disorders. The twenty years that elapse between the publications of the two different editions are marked by the failure of microbiology to identify genetic (multigenetic) causes of autism. This consideration has led to a significant epistemological change within the classification of “autisms”; first of all, the trick of inventing a “spectrum” of autism can include diagnoses close to autistic disorders, subthreshold diagnoses such as PDD-NOS or diagnoses that differed only in intellectual abilities, such as Asperger Syndrome.

The literature review carried out through this paper demonstrated the high comorbidity between PDD-NOS and AD before DSM-5, and then with ASD, particularly during adolescence. The impossibility of making a differential diagnosis between the two, we believe, has made it necessary to revisit this diagnostic category and the construction of a new umbrella category, such as the autism spectrum. In fact, the removal of PDD-NOS has certainly generated a great deal of confusion and has had to consider the accounts with the social prejudice against the diagnosis of autism that terrified parents in the 1990s and 2000s. The diagnosis of PDD-NOS made the parents breathe a sigh of relief; the latter then found themselves seeing the diagnosis of PDD-NOS disappear and having to consider their son as having a disorder attributable to autism. This has thrown many parents into panic, who had for years thought and spent energy in building intervention plans linking them to possible causes and to the functioning characteristics of PDD-NOS.

This study closes with the consideration of making the diagnostic processes more intelligible, bringing out the interpretative dimension at the basis of the construction of the diagnosis itself, a process which, if made explicit, can help the interlocutors to trace developmental objectives for the diagnoses of autism without having to fall back in search of underlying causes of neurodevelopmental disorders, which are known to be multifactorial.

Given the severity of the conditions that the autism spectrum entails, and the impairment in most cases of autonomy processes, the psycho-pedagogical and educational element of intervention remains a milestone within which it is worth turning the interest of public health policy [31,32].

## 7. Research Limitations, Future Directions and Policy Implications

This study has several limitations that are particularly relevant and of which the reader should be aware of. The first limitation is the presence of a scarce reference literature, probably due to the elimination of this specific diagnosis in the latest edition of the DSM-5. This has in fact considered PDD-NOS an outdated diagnosis. There is also a lack of studies that trace the confusion on the part of families and the professional community to see this diagnosis disappear. The second limit concerns the fact that the studies included in the review used small samples of individuals, and moreover, many of these use qualitative criteria to make the inferences. This is also attributable to the fact that the deletion of the diagnosis of PDD-NOS from DSM 5 has not stimulated further research in this regard, nor has it aroused interest in both national and international health organizations in carrying out large-scale epidemiological research. Future studies should consider the following: (1) the search for any divergent characteristics of functioning (the same and different) between adults who received a PDD-NOS diagnosis and those who received AD diagnoses in childhood; (2) the collections of the point of view and experience of psychiatrists and neuropsychiatrists on the usefulness of a subthreshold diagnosis such as PDD-NOD compared to an umbrella category such as the ASD and its clinical implications; (3) collect the voice of support teachers on the degree of impairment in learning of pupils with PDD-NOD and on the compensatory strategies used, including the Augmentative Alternative Communication.

From the point of view of clinical implications and for policies, we are currently witnessing a depletion of public health resources aimed at supporting community mental health [33] and fragile generations [34], as well including minority groups [35,36,37].This has generated a strong emergence and organization of non-profit organizations that are involved in accompanying the development of children with ASD towards adulthood and in training educators and assistants to carry out home and educational interventions. Surely in the coming years, the increase in ASD diagnoses will have to correspond to an increase in resources aimed at a professional retraining of health care professionals and the use of integrated psychological methods of intervention that take into account the need to support families.

## Figures and Tables

**Table 1:** Database search indicators.

Article screening strategy	KEYWORDS: (PDD-NOS[Title/Abstract]) OR (pervasive developmental disorder not otherwise specified[Title/Abstract])) NOT (asperger[Title/Abstract])) AND (diagnosis[Title/Abstract])) AND (ENGLISH[Language]). Timespan: from January 2010 up to February 2023. Electronic Databases: PubMed, Scopus, PsychINFO

**Table 2:** Study characteristics and results.

Author, Year	Type	Sample Size	Technique
1. Kozlowski, 2010 [10]	Diagnostic differences between PDD-NOS and ASD	322 infants from 17 to 36 months	BISCUIT score
2. Hattier and Matson, 2012 [11]	Communication deficits: differences between PDD-NOS and ASD	591 infants from 17 to 36 months	BISCUIT part 1 score
3. Louwerse et al., 2015 [12]	Comorbidity between ASD and PDD-NOS	72 individuals with PDD-NOS diagnosis	ADOS, DISC, FSIQ, TRAILS, ADI-R
4. Gibbs et al., 2012 [13]	PDD-NOS diagnosis comparison between DSM-IV-TR and DSM-V	132 children	-
5. Horovitz and Matson, 2010 [14]	Communication deficits: differences between PDD-NOS and ASD	-	BISCUIT part 1 score
6. Matson et al., 2010 [15]	Comorbidity between ASD and PDD-NOS	-	BISCUIT part 2 score
7. Snow and Lecavalier, 2011 [7]	Diagnostic differences between PDD-NOS and ASD	-	-
8. Brennan et al., 2017 [5]	PDD-NOS diagnosis criteria	160 children: 102 (Age 2) and 58 (Age 4)	ADOS, ADI, CARS scores
9. Carigi et al., 2014 [16]	Diagnostic differences between PDD-NOS and ASD	117 children from 17 to 175 months	CARS, BSE, GMDS, VABS, CGI, CBCL, EuroQol
10. Rooney et al., 2011 [17]	PDD-NOS differential diagnosis and prognosis	Case study (Age 7)	WISC-IV, ADIS-C/P YBOCS-C, SUDS
11. Mayes et al., 2013 [18]	PDD-NOS diagnosis comparison between DSM-IV-TR and DSM-V	-	-
12. Matson et al., 2011 [19]	Comorbidity between ASD and PDD-NOS	362 infants …	BISCUIT part 2, BISCUIT part 3 score
13. Hassan and Perry, 2011 [20]	PDD-NOS diagnosis criteria	105 children from 2 to 12 years	VABS, VABS- II, MSEL, BSID, WPPSI-3, CARS, Leiter-R, Stanford-Binet Intelligence Scale
14. Taheri and Perry, 2012 [21]	PDD-NOS diagnosis criteria	131 children from 2 to 12 years	
15. Karabekiroglu and Akbas, 2011 [22]	PDD-NOS differential diagnosis	188 children from 2 to 11 years	K-SADS-PL-T, PDD-NOS Symptom Screening Scale, ADI
16. Andrews et al., 2013 [23]	PDD-NOS and ASD prognosis	58 children from 7 to 12 years	
17. Mordre, 2012 [24]	PDD-NOS and ASD prognosis	113 children from	
18. Rondeau et al., 2011 [25]	PDD-NOS diagnosis criteria	-	-
19. Matson and Sipes, 2010 [26]	PDD-NOS diagnosis criteria	-	-
20. Romero et al., 2016 [27]	Comorbidity between ASD and PDD-NOS	123 children from 5 to 15 years	NCBRF, IDEA
21. Kose et al., 2017 [28]	PDD-NOS prognosis	51 children from 2 to 16 years	CARS, ABC
22. Verheij, 2015 [29]	Comorbidity between ASD and PDD-NOS	74 children from 6 to 12 years	DISC-IV-P, CSBQ, IQ, YSR
23. Mandy, 2011 [30]	PDD-NOS diagnosis criteria	256 children	-

## Data Availability

Data will be made available upon reasonable request to the corresponding author.
